# Transgenic Expression of Nonclassically Secreted FGF Suppresses Kidney Repair

**DOI:** 10.1371/journal.pone.0036485

**Published:** 2012-05-14

**Authors:** Aleksandr Kirov, Maria Duarte, Justin Guay, Michele Karolak, Cong Yan, Leif Oxburgh, Igor Prudovsky

**Affiliations:** 1 Maine Medical Center Research Institute, Maine Medical Center, Scarborough, Maine, United States of America; 2 Department of Pathology, University of Indiana, Indianapolis, Indiana, United States of America; Institut National de la Santé et de la Recherche Médicale, France

## Abstract

FGF1 is a signal peptide-less nonclassically released growth factor that is involved in angiogenesis, tissue repair, inflammation, and carcinogenesis. The effects of nonclassical FGF export in vivo are not sufficiently studied. We produced transgenic mice expressing FGF1 in endothelial cells (EC), which allowed the detection of FGF1 export to the vasculature, and studied the efficiency of postischemic kidney repair in these animals. Although FGF1 transgenic mice had a normal phenotype with unperturbed kidney structure, they showed a severely inhibited kidney repair after unilateral ischemia/reperfusion. This was manifested by a strong decrease of postischemic kidney size and weight, whereas the undamaged contralateral kidney exhibited an enhanced compensatory size increase. In addition, the postischemic kidneys of transgenic mice were characterized by hyperplasia of interstitial cells, paucity of epithelial tubular structures, increase of the areas occupied by connective tissue, and neutrophil and macrophage infiltration. The continuous treatment of transgenic mice with the cell membrane stabilizer, taurine, inhibited nonclassical FGF1 export and significantly rescued postischemic kidney repair. It was also found that similar to EC, the transgenic expression of FGF1 in monocytes and macrophages suppresses kidney repair. We suggest that nonclassical export may be used as a target for the treatment of pathologies involving signal peptide-less FGFs.

## Introduction

Members of the fibroblast growth factor (FGF) family play critical roles in developmental and pathological processes [1], [2], [3], [4]. Most FGFs signal through specific transmembrane receptors (FGFR), and thus require secretion for their biological activities [1], [2], [3], [4]. The majority of FGFs have a cleavable hydrophobic N-terminal peptide in their structure that allows their release through the classical secretion pathway, which involves the endoplasmic reticulum and Golgi apparatus. In contrast, two most widely expressed members of the family, FGF1 and FGF2, are devoid of signal peptide and thus are released through unconventional secretion pathways [5], [6], [7], [8]. FGF1 and FGF2 are expressed in epithelial and endothelial cells, and mononuclear leukocytes in kidneys under normal and pathological conditions [9], [10], [11], [12]. FGF1 has potent effects in embryonic kidney culture, regulating ureteric bud branching [13] and nephron progenitor cell maintenance [14]. Recombinant FGF1 and FGF2 improve wound healing [15], post-ischemic heart repair [16], [17], [18], [19], and formation of collaterals after hindlimb ischemia [20]. The knockdown of FGFR2 exacerbated [21] and delivery of recombinant FGF2 attenuated [22] postischemic kidney damage. However the latter experiments were limited to first 4 days after ischemia apparently due to insufficient stability of recombinant FGFs in the organism [23].

We propose to use conditional transgenic expression of FGF1 to elucidate long-term effects of this signaling ligand on kidney injury. To that end, we created transgenic mice with conditional FGF1 expression in endothelial cells (EC) abundantly present in kidneys. The production of FGF1 in EC directly facing the bloodstream facilitated the assessment of its release. Another advantage of our in vivo model was that despite permanent FGF1 expression in EC, transgenic animals exhibited a normal phenotype, including unperturbed kidney structure; therefore, we were able to specifically focus on FGF-dependent events caused by ischemia and postischemic stress. We found that transgenic expression of FGF1 in EC resulted in the irreversible loss of epithelial tubular structures and massive fibrosis in the postischemic kidney. Importantly, these effects were suppressed by taurine, which inhibits nonclassical FGF1 export in vitro [24] and also in vivo as we found in the present study. These data demonstrate that transgenic expression of nonclassically released FGF is compatible with normal development and morphology of kidneys, but it suppresses postischemic kidney repair. In addition, they suggest that targeting nonclassical FGF export may be used for the treatment of pathological conditions caused by naturally occurring upregulation of FGF1 and FGF2 expression [25], [26], [27].

## Materials and Methods

### 1. Production of Transgenic Mice

To produce transgenic mice, we used FGF1 with a R136K mutation at the thrombin cleavage site located in the heparin binding domain of FGF1. R136K FGF1 developed as an agent for wound repair exhibits normal mitogenic activity [28], and we demonstrated that similar to wild type FGF1, it is normally released during cell stress [29]. The use of this mutant prevented thrombin cleavage of FGF1 and thus assured the efficient collection of vascular FGF1. R136K mutation was introduced by PCR-based site-directed mutagenesis in the FGF1pMEXneo expression construct [5]. The codon encoding arginine 136 (AGA) was changed to a lysine (AAA) (FGF1R136K). FGF1R136K was cloned into *SalI* and *EcoRI* restriction sites of pcDNA3-HA vector originating FGF1R136K:HA-pcDNA3. We inserted FGF1R136K:HA into the *HindIII* and *XbaI* restriction sites of the pTRE-Tight expression vector (Clontech) to obtain the FGF1R136K:HA-pTRE construct. Pronuclei of fertilized oocytes of FVB mice (Taconic) were injected with FGF1R136K:HA-pTRE-Tight DNA. RT-PCR was applied to test the integration of the transgene in the genome of one-month-old mice produced from injected oocytes. The following two pairs of primers were used:

- 5′-CGT GTA CGG TGG GAG GCC-3′ and 5′- CAA ATG TGG TAT GGC TGA TT- 3′
- 5′-GGC TCA CAG ACA CCA AAT G-3′ and 5′- CAA ATG TGG TAT GGC TGA TT- 3′

FGF1-positive mice were bred with FVB partners; as a result, six independent transgenic lines were obtained.

To check the inducibility of FGF1 expression in transgenic mice, we analyzed cultures of tail fibroblasts. The tail snips from FGF1-transgenic mice were sterilized with ethanol, the skin was removed, and snips were digested using 70 units/ml solution of collagenase (Sigma) in DMEM for 3 h at 37°C. Large cell clumps were then allowed to sediment at 1 g for 5 min. Dissociated cells were precipitated by centrifugation at 2000 g, resuspended in DMEM plus 10% fetal calf serum, and plated on fibronectin-coated glass coverslips placed in the wells of TC6 plates. One week later, the cells were transiently transfected using Fugene (Roche) with the CMVt-rtTA construct (gift of Dr. John Hiscott, McGill University, Montreal, Canada). One day after rtTA transfection, the fibroblasts were stimulated with 10 µg/ml doxycycline (Sigma), incubated for an additional 48 h, fixed with 10% formalin, and immunofluorescently stained with mouse anti-HA antibodies (Covance) followed by FITC-conjugated anti-mouse IgG antibodies (Vector Laboratories), and examined under a confocal fluorescence microscope. Fibroblasts untransfected with rtTA or transfected with rtTA but untreated with doxycycline did not express FGF1:HA. Of the six FGF1 transgenic lines examined, line F demonstrated the most reproducible rtTA-dependent and doxycycline inducible expression of FGF1:HA. Mice of this line were bred with Tg(Tek-rtTA,TRE-lacZ)1425Tpr/J transgenic mice expressing rtTA under the EC-specific Tek promoter (The Jackson Laboratory). The double transgenic mice were further bred for three generations onto the FVB background before using them in kidney ischemia experiment. The genotype of double transgenic FGF1/Tek mice was confirmed by RT-PCR using the above mentioned FGF1 primers and two following combinations of Tg(Tek-rtTA,TRE-lacZ)1425Tpr/J-specific primers recommended by The Jackson Laboratory:

- 5′-CAA ATG TTG CTT GTC TGG TG-3′; 5′-GTC AGT CGA GTG CAC AGT TT-3′; 5′-CGC TGT GGG GCA TTT TAC TTT AG-3′; 5′-CAT GTC CAG ATC GAA ATC GTC-3′.
- 5′-CAA ATG TTG CTT GTC TGG TG-3′; 5′-GTC AGT CGA GTG CAC AGT TT-3′; 5′-ATC CTC TGC ATG GTC AGG TC-3′; 5′-CGT GGC CTG ATT CAT TCC-3′.

In parallel, we bred FGF1 transgenic mice with the animals expressing rtTA under the macrophage/monocyte specific c-fms promoter. The original rtTA/c-fms transgenic mice [30] were bred onto the FVB background before being mated with FGF1 transgenic animals. The genotype of double transgenic FGF1/c-fms animals was confirmed by PCR using the aforementioned FGF1 primers and a pair of rtTA/c-fms primers:

5′ – TGA TTG AAG GGT CCA GAC TCA TTC –3′ and 5′ – AGT GTA GGC TGC TCT ACA CCA AGC –3′

### 2. Determination of FGF1 Content in the Vasculature

To determine the content of FGF1 in the vasculature, the mice were sacrificed by isoflurane inhalation and their cardiovascular system was immediately perfused with 10 ml of ice-cold PBS containing 10 units/ml heparin (Sigma) in order to detach FGF1 bound to endothelial cells (EC). The diluted blood was collected and centrifuged for 10 min at 700 g. The content of FGF1 in the supernatant was determined using the FGF1 ELISA kit (R&D Systems). To determine the FGF1 content in the vasculature, we considered that the blood volume in mice averages 7% of its body weight, and assumed that all the blood was collected by perfusion.

### 3. Determination of FGF1 Content in Kidneys

To detect FGF1 expression in kidneys, the mice were sacrificed by isoflurane inhalation followed by cervical dislocation, and a 50 mg fragment of the kidney was snap frozen in liquid nitrogen, pulverized, and lyzed in SDS-PAGE loading buffer. Samples were resolved by 15% SDS-PAGE and immunoblotted using rabbit anti-FGF1 antibodies.

### 4. Unilateral Kidney Ischemia Reperfusion Injury

This study has been performed in accordance with the National Research Council *Guide for the Care and Use of Laboratory Animals* and approved by the Institutional Animal Care and Use Committee of Maine Medical Center (approval ID 0701). Only male animals were used in all the experiments. Male mice between 2 and 3 months of age were anesthetized using a cocktail of xylazine (14.5 µg/kg) and ketamine (95 µg/kg), and maintained at a core temperature of 37°C for the duration of the surgical procedure. The renal pedicle of the right kidney was clamped for 26 min using a microaneurysm clamp, during which time the kidney was retained in the abdominal cavity. Animals in which the kidney either did not discolor within one min after clamping or return to normal color within two min after removal of the clamp were excluded from the experiment. Following release of the clamp, the kidney was returned to the abdomen and the body wall was sutured to prevent prolapse. Skin staples were used to close the wound.

### 5. RNA Purification and qRT-PCR Analysis

Isolated kidney tissue was added to 1 ml Trizol (Invitrogen) on ice, homogenized immediately, and snap frozen. Crude total RNA was purified from 500 µl of lysate according to the manufacturer’s instructions, and further purified using the RNeasy Mini kit (Qiagen) with DNase treatment. One µl of Ribolock (Fermentas) was added, and cDNA was generated from 1 µg of RNA using the qScript cDNA kit (Quanta). For qPCR, 1 µl of cDNA was used as template in a 25 µl reaction using iQ SYBR Green SuperMix (BioRad) on a MyiQ real time detection system (Biorad). Cycling parameters were 95°C for 15 seconds or 55°C for 45 seconds. Primer sequences for mouse *KIM1* (*Havcr1*) were 5′-TCGTGTCACCTATCAGAAGAGC-3′ and 5′- ACAATACAGACCACTGTCACTC-3′, and for the β-actin housekeeping gene 5′- CGTGCGTGACATTAAAGAGAAG-3′ and 5′- TGGATGCCACAGCATTCCATA-3′. Each biological sample was assayed in triplicate. Technical replicates were averaged and 1/ΔCT was calculated for each biological sample. The SEM was determined within each group of biological replicates, and p values were calculated using the Student’s *t*-test.

### 6. Histology and Immunohistochemistry

After perfusion of the mouse vascular system with ice-cold heparinized PBS, the postischemic and control contralateral kidneys were fixed for 24 h in the cold 10% neutral formalin. The paraffin sections of kidneys were prepared and stained with hematoxylin and eosin, Trichrome, Sirius Red or periodic acid-Schiff stain (PAS). For DAB-immunoperoxidase detection of transgenically expressed FGF1, we used anti-HA monoclonal antibodies (Covance). To identify EC, macrophages, neutrophils, and proliferating cells, we used respectively: (i) anti-CD31/PECAM biotinylated rat antibodies (BD Pharmingen), (ii) F4/80 mouse monoclonal antibodies (Santa Cruz), (iii) rat anti-neutrophil antibodies (AbD Serotec), and (iv) anti-Ki-67 rabbit antibodies (Abcam).

### 7. Image Analysis and Statistics

Kidney tubules were counted on hematoxylin and eosin stained sections, at magnification ×10, in 10 fields per section, by using MacBiophotonic ImageJ program. Decrease of tubule density was presented as the ratio (%) of average tubular density in postischemic kidneys to that in contralateral kidneys. From four to nine mice per experimental group were studied. Means and SEM were calculated by using GraphPad software. The significance of differences between transgenic and wild type (WT) animals was assessed using Student’s t-test. The same imaging and statistical analysis programs were used to assess the portion (%) of kidney section positive for Sirius Red staining (magnification ×40, Sirius Red and hematoxylin staining), percentage of Ki-67-positive nuclei (magnification ×20, immunoperoxidase staining, hematoxylin counterstaining), and percentage of dilated tubules, i.e. those where lumen occupies more than ¼ of tubule section (magnification ×20, hematoxylin and eosin staining). Increase of tubule dilatation was presented as the ratio (fold) of the percentage of dilated tubules in the postischemic kidney to that in the contralateral kidney. Measurement of glomeruli diameters was performed on hematoxylin and eosin stained sections at magnification ×20, by using a micrometer stage.

## Results

### 1. FGF1/Tek Transgenic Mice Stably Express FGF1 in EC and Release it to the Circulation

**Figure 1:**
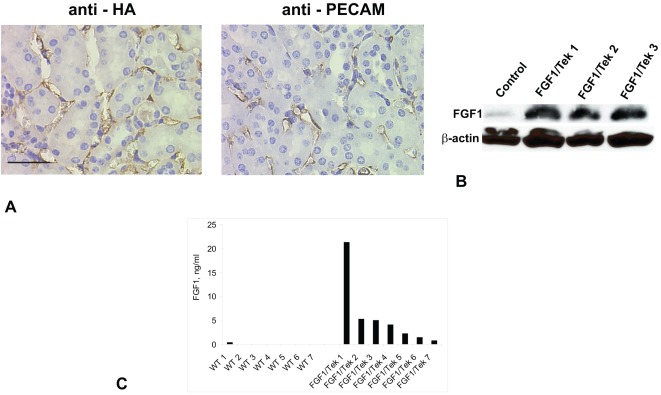
Transgenic FGF1 expression and release in FGF1/Tek mice. A. FGF1 is expressed in kidney EC. Immunoperoxidase staining was used to detect transgenic FGF1 (anti-HA antibodies) and EC (anti-PECAM antibodies) in the paraffin sections of kidneys obtained from FGF1/Tek mice. Preparations were counterstained with hematoxylin. Bar –30 µ. B. Lysates of kidney tissue obtained from FGF1/Tek and control FVB mice were resolved by SDS-PAGE and immunoblotted using rabbit anti-FGF1 antibodies and mouse monoclonal anti-ß-actin antibodies (loading control). C. Transgenically expressed FGF1 is released into the vasculature of FGF1/Tek mice. Seven male FGF1/Tek mice and 7 control WT FVB males were sacrificed; their vasculatures were perfused with cold heparinized PBS and the content of FGF1 (ng/ml blood) was determined using an FGF1 ELISA kit from R&D.

To assess the effect of EC-derived FGF1 on postischemic kidney repair, we produced transgenic mice with EC-specific expression of human FGF1. C-terminal HA tag, which does not interfere with FGF1 release [24], was used for immunohistochemical FGF1 detection. To ensure cell type specific FGF1 expression, we chose a Tet-based system. FGF1 was cloned in the pTRE-Tight plasmid (Clontech) and the resultant construct was injected into fertilized mouse oocytes. We produced several independent lines of FGF1 transgenic FVB mice. By immunoblotting or immunohistochemistry, none were found to express detectable amounts of FGF1:HA in kidney, muscle, or liver (data not shown). To assess the potential of rtTA-dependent FGF1 expression in transgenic mice, we isolated fibroblasts from their tails and transiently transfected them with rtTA. Immunofluorescence analysis demonstrated that doxycycline treatment induced FGF1:HA expression in cultured mouse fibroblasts derived from transgenic mice (data not shown). We bred pTRE-Tight/FGF1 transgenic mice with Tg(Tek-rtTA, TRE-lacZ)1425Tpr/J transgenic mice expressing rtTA under the EC-specific Tek promoter. Double transgenic FGF1/Tek mice were bred onto the FVB background. Immunoperoxidase staining demonstrated endothelium-specific expression of FGF1:HA in kidneys without doxycycline treatment (Figure 1A). Western immunoblot analysis confirmed the expression of FGF1 in the kidneys of the FGF1/Tek mice dramatically exceeding endogenous FGF1 level (Figure 1B). Furthermore, FGF1 was detected by ELISA in the vasculature of FGF1/Tek mice untreated with doxycycline but not in the control mice (Figure 1C). FGF1/Tek mice did not display any visible pathology and had normal fertility. The kidneys of FGF1/Tek animals were indistinguishable from those of control animals by their macroscopic ad microscopic morphology. The unexpected stable uninduced EC-specific expression of FGF1 in FGF1/Tek mice simplified our experiments by eliminating the need for doxycycline stimulation.

### 2. Early Postischemic Response in FGF1/Tek and Control Animals

**Figure 2:**
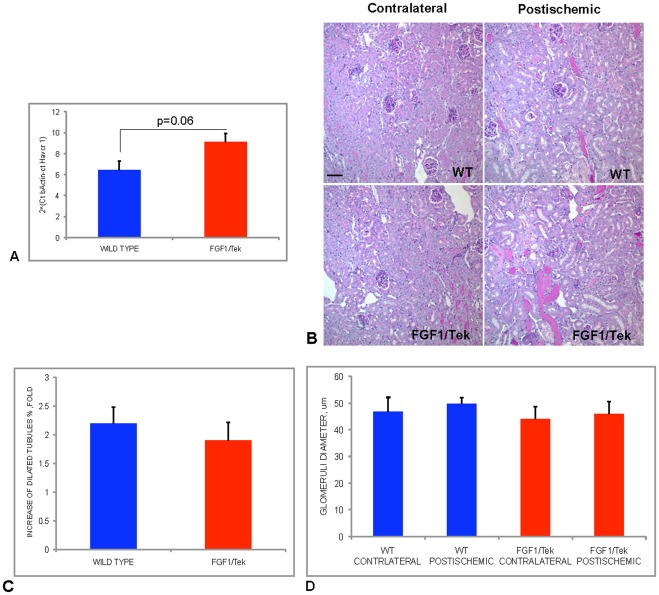
Early postischemic response in FGF1/Tek and control animals. A. Expression of the kidney injury marker 1, Havcr1, in the postischemic kidneys of FGF1/Tek and control WT FVB mice one day after ischemia. qRT-PCR results normalized to ß-actin expression. Kidneys of five FGF1/Tek and five WT mice were studied. Mean and SEM are presented. B. PAS staining of the paraffin sections of postischemic and contralateral kidneys of control WT FVB and FGF1/Tek mice. One day after ischemia, hematoxylin counterstaining. Bar –40 μ C. Increase of the percentage of tubules with enlarged lumen (lumen occupies more than ¼ of tubule section) in postischemic kidneys comparatively to contralateral organs. Kidneys of four FGF1/Tek and four WT mice were studied. Mean and SEM of fold increase are presented. D. Glomeruli diameters in postischemic and contralateral kidneys. Kidneys of four FGF1/Tek and four WT mice were studied. Mean and SEM are presented.

Two- to three-month-old male FGF1/Tek transgenic mice and control male FVB mice were subjected to 26 min of transient ischemia of the right kidney. Day 1 after surgery, the mice were sacrificed and the level of the mouse ortholog of kidney injury marker 1 (KIM1), *Havcr1,* was determined by qRT-PCR. One day after ischemia, the expression of *Havcr1* in FGF1/Tek animals was higher than in control mice (Figure 2A). Despite the trend of increased *Havcr1* expression in transgenic mice relative to WT, differences between the two genotypes did not reach p<0.05. PAS staining of kidney sections demonstrated that 24 h after ischemia, the postischemic kidneys of both FGF1/Tek and control animals contained in contrast to contralateral organs epithelial tubules, filled with PAS-positive protein casts characteristic of acute kidney injury (Figure 2B). Postischemic kidneys were characterized by an increase of the percentage of dilated tubules, and this increase was not significantly different between FGF1/Tek and control animals (Figure 2C). Ischemia did not result in a significant change of the diameters of glomeruli in the kidneys of both FGF1/Tek and control mice (Figure 2D).

### 3. Kidney Ischemia/Reperfusion in FGF1/Tek Mice Results in Size Decrease of Postischemic Kidneys and Loss of Tubular Structures

**Figure 3:**
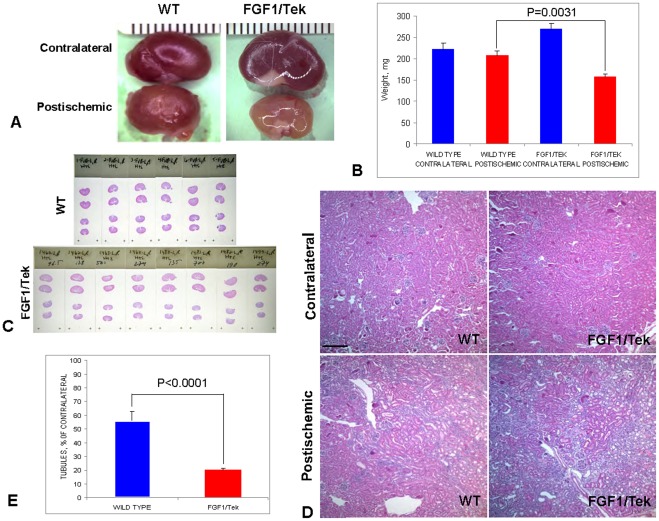
Decrease of postischemic kidney size and loss of tubular epithelial structures in FGF1/Tek animals. A. Representative contralateral and postischemic kidneys of FGF1/Tek and control FVB (WT) animals, 21 days after ischemia/reperfusion. B. Decrease of postischemic kidneys weight (red) and increase of contralateral kidneys weight (blue) in FGF1/Tek mice compared to FVB mice (WT). Mean and SEM are presented. C. Sections of contralateral (two top sections per slide) and postischemic (two bottom sections per slide) kidneys of FGF1/Tek and control mice. D. Loss of tubular structures in a postischemic kidney of an FGF1/Tek mouse. Postischemic and contralateral kidneys of an FGF1/Tek and a control WT mouse are presented. Hematoxylin/eosin stained paraffin sections. Bar −120µ. E. Postischemic/contralateral % ratio (mean and SEM) of kidney tubule density in FGF1/Tek and WT mice. Numbers of epithelial tubular structures in ten ×10 objective field were counted in postischemic and contralateral kidneys of four FGF1/Tek and four wild type mice.

Three weeks after unilateral ischemia/reperfusion, postischemic kidneys of FGF1/Tek animals presented a sharp morphological contrast to control animals. Indeed, their weight was on average 25% less than in the control mice (Figure 3A,B,C). Hematoxylin-eosin staining of paraffin sections revealed a failure of kidney repair in postischemic FGF1/Tek animals. Indeed, three weeks after surgery, the postischemic kidneys of FGF1/Tek mice exhibited a paucity of epithelial tubules, combined with hyperplasia of interstitial cells (Figure 3D,E). Conversely, the histology of postischemic kidneys in control WT animals was more similar to that of contralateral organs: efficient restoration of tubular structures, and few signs of interstitial hyperplasia. The ratio of tubule density in postischemic FGF1/Tek mice kidneys to tubule density in contralateral kidneys (Figure 3E) was sharply lower than this parameter in WT mice. The postischemic kidneys of FGF1/Tek animals contained abundant capillaries detected by anti-PECAM staining (Figure S1). Thus, it is unlikely that the observed changes were due to deficient angiogenesis.

### 4. Ischemia Results in Fibrosis of FGF1/Tek Kidneys

**Figure 4:**
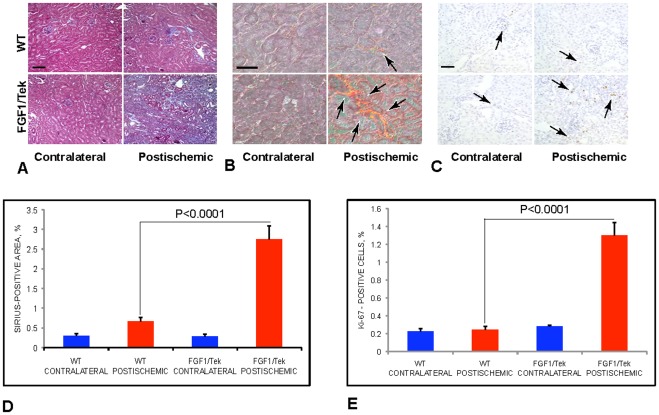
Fibrosis and cell proliferation in the postischemic kidneys of FGF1/Tek mice. Twenty-one days after ischemia, paraffin sections of postischemic and contralateral kidneys of FGF1/Tek and control FVB mice. A. Trichrome staining. B. Sirius red staining for collagen. Polarization microscopy. C. Immunoperoxidase staining for Ki-67, a marker of cell proliferation. In B and C, hematoxylin counterstaining was used. Bar in A –80 µ. Bars in B and C –40 µ. D. Quantification of Sirius staining: % of kidney section area positive for Sirius Red (mean and SEM) is presented. Kidneys of four FGF1/Tek and four WT mice were studied. E. Quantification of cell proliferation: % cells positive for Ki-67 (mean and SEM) is presented. Kidneys of four FGF1/Tek and four WT mice were studied.

Abundance of interstitial cells in postischemic kidneys of FGF1/Tek mice prompted us to assess fibrosis and cell proliferation in these organs. Trichrome staining (Figure 4A) revealed a strong expansion of connective tissue in the cortex and medulla of postischemic kidneys of FGF1/Tek mice compared to control animals. Staining with the collagen marker Sirius Red was increased in the interstitium of postischemic FGF1/Tek kidneys much stronger than in postischemic kidneys of control mice (Figure 4B, D). Immunohistochemical staining for Ki-67 a marker of cell proliferation, revealed numerous proliferating cells in the hyperplastic interstitium of cortex and medulla of FGF1/Tek kidneys, while Ki-67-positive cells were rare in postischemic kidneys of control animals (4C, E).

### 5. FGF1 Expression in EC Enhances the Invasion of Macrophages and Neutrophils into the Postischemic Kidneys

**Figure 5:**
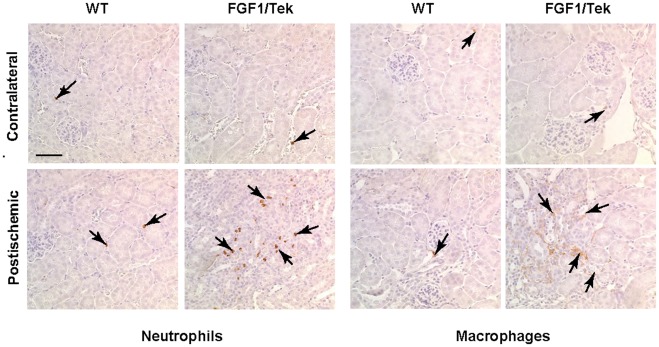
Massive infiltration of neutrophils and macrophages in the postischemic kidneys of FGF1/Tek mice. Twenty-one days after ischemia, paraffin sections of the postischemic and contralateral kidneys of FGF1/Tek and control FVB mice were stained using the immunoperoxidase method for a neutrophil marker or F4/80, a macrophage marker. Hematoxylin counterstaining. Bar –40 μ.

Kidney fibrosis is enhanced by the invasion of macrophages and neutrophils that serve as sources of various profibrotic growth factors and cytokines [31], [32]. To assess the effect of EC-derived FGF1 on the invasion of inflammatory cells into the kidneys, we used immunoperoxidase histochemistry with the antibody F4/80 (marker of macrophages) or an anti-neutrophil antibody. We found that three weeks after surgery, postischemic FGF1/Tek kidneys contained large groups of macrophages and neutrophils in the interstitium, whereas in the postischemic kidneys of control animals, only individual macrophages and neutrophils were found (Figure 5). Contralateral kidneys of both FGF1/Tek and control animals were also largely macrophage- and neutrophil-negative.

### 6. Taurine Inhibits FGF1 Export in vivo and Rescues Kidney Repair in FGF1/Tek Mice

Several pharmacological agents have been used with variable success to treat kidney ischemia and fibrosis [33], i.e. antioxidants, NO inhibitors, erythropoietin, adenosine, and others [33]. One of the agents with a beneficial effect on postischemic renal recovery is the sulfur-containing non-essential amino acid, taurine [34].

In addition to its anti-oxidant properties, taurine acts as a membrane stabilizer [34]. Because FGF1 is an acidic phospholipid-binding protein [35] that destabilizes membranes containing acidic phospholipids [36], we hypothesized that taurine could inhibit FGF1 export in transgenic animals. Indeed, our recent study [24] showed that in vitro stress-induced FGF1 export correlates with phosphatidylserine externalization, and both processes are inhibited by taurine. Therefore, we assessed the effect of taurine on postischemic kidney repair and FGF1 export in FGF1/Tek mice. The mice were administered water containing 10 mg/ml taurine from 48 h before to 21 days after kidney ischemia, when the taurine-treated and control FGF1/Tek animals were sacrificed to obtain kidneys and blood samples. Taurine treatment resulted in a repression of FGF1 export to circulation in most mice (Table 1). We also observed that while the weight of postischemic kidneys in the six control animals varied between 168 and 187 mg, in the four out of six mice treated with taurine, it was between 201 and 217 mg (Table 1). Unlike control animals, these four taurine treated mice contained no detectable FGF1 in the vasculature (Table 1). Taurine did not inhibit FGF1 export in two animals, and these mice had low postischemic kidney weights: 186 and 180 mg (Table 1). Thus, the rescuing effect of taurine on postischemic kidney repair correlated with the suppression of FGF1 export.

**Table 1:** Effect of taurine treatment on FGF1 release and postischemic kidney weight in FGF1/Tek mice.

Mouse #	Taurine treatment	Contralateral kidney weight, mg	Postischemic kidney weight, mg	FGF1 blood content, ng/ml
1	−	242	174	5.32
2	−	201	168	1.52
3	−	233	187	3.66
4	−	203	184	2.38
5	−	250	182	4.75
6	−	296	171	4.27
1t	+	231	217	0
2t	+	240	201	0
3t	+	210	201	0
4t	+	229	203	0
5t	+	225	186	6.61
6t	+	304	180	0.86

FGF1/Tek mice were fed with water containing taurine (10 mg/ml) or taurine-free water from 2 days before to day 21 after ischemia/reperfusion, when they were sacrificed. FGF1 content in the vasculature was determined by the ELISA method.

**Figure 6:**
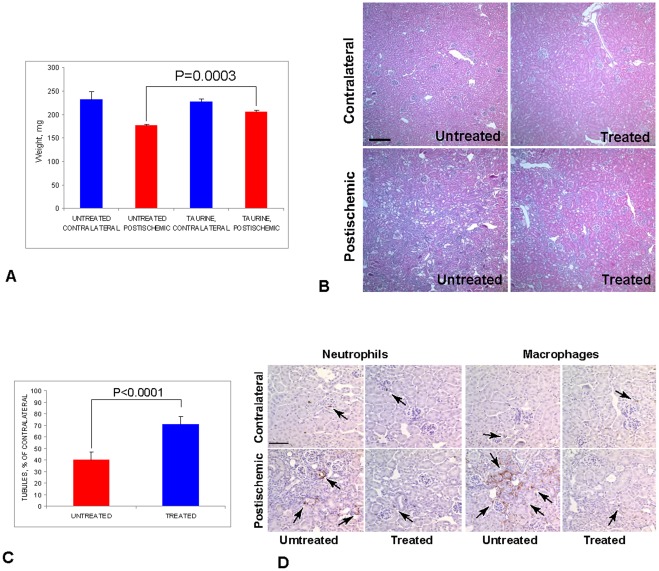
Taurine inhibits FGF1 release in FGF1/Tek mice and rescues the postischemic kidney recovery. FGF1/Tek mice were fed with water containing taurine (10 mg/ml) or taurine-free water from 2 days before to day 21 after ischemia/reperfusion, when they were sacrificed. FGF1 content in the vasculature was determined by the ELISA method (see Table 1). Four taurine-treated mice with inhibited FGF1 export and six untreated mice were studied. A. Weights of contralateral and postischemic kidneys. Means and SEM are presented. B. Representative hematoxylin/eosin stained paraffin sections of the postischemic and contralateral kidneys of a taurine-treated mouse and a control mouse. Bar −120µ. C. Postischemic/contralateral % ratio (mean and SEM) of kidney tubule density in taurine-treated and untreated mice. Numbers of epithelial tubular structures in ten ×10 objective fields were counted in postischemic and contralateral kidneys of four FGF1/Tek mice with taurine-inhibited FGF1 release and six FGF1/Tek mice untreated with taurine. D. Paraffin sections of the postischemic and contralateral kidneys of taurine-treated and untreated mice were stained using the immunoperoxidase method for a neutrophil marker or F4/80, a macrophage marker. Hematoxylin counterstaining. Bar –40 µ.

Besides having significantly increased weight (Figure 6A), postischemic kidneys of mice with FGF1 release repressed by taurine had less interstitial hyperplasia than the postischemic kidneys of animals not treated with taurine (Figure 6B). The postischemic/contralateral ratio of kidney tubule density in mice with repressed FGF1 release (Figure 3C) was almost twice higher than in FGF1/Tek animals untreated with taurine. In addition, unlike taurine untreated animals, mice with FGF1 release repressed with taurine did not exhibit large groups of macrophages and neutrophils in the interstitium of their postischemic kidneys (Figure 3D).

### 7. Similar to FGF1/Tek Animals, FGF1/c-fms Transgenic Mice Exhibit Attenuated Postischemic Kidney Repair

**Figure 7:**
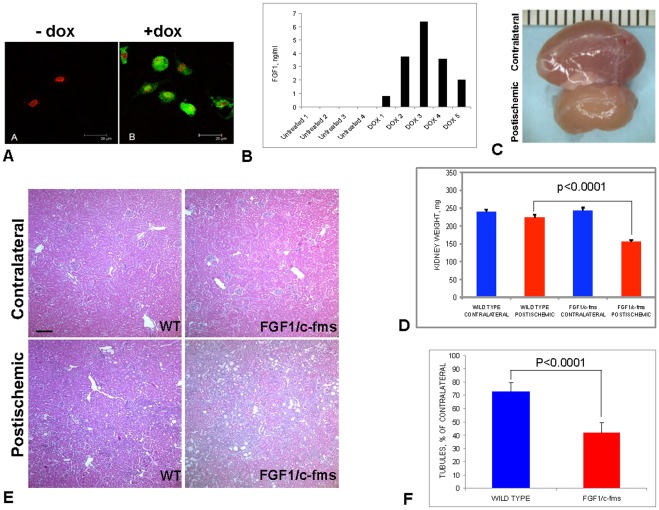
Decrease of organ size and loss of tubular structures in the postischemic kidneys of FGF1/c-fms transgenic mice. A. Induction of FGF1/HA expression in the peritoneal macrophages of FGF1/c-fms transgenic mice. Forty-eight hours before being sacrificed, the animals were intraperitoneally injected with 0.2 ml PBS containing 10 µg/ml doxycycline (right) or with doxycycline-free PBS (left). Macrophages were obtained by flushing the peritoneal cavity with PBS, plated on coverslips in DMEM with 10% FBS, fixed 12 h after plating and stained using anti-HA antibodies (green) and TOPRO3 (red). Confocal images are presented. Bar –20µ. B. FGF1 release in the vasculature of FGF1/c-fms mice. The animals were intraperitoneally injected with 0.2 ml PBS containing 10 µg/ml doxycycline (right) or with doxycycline-free PBS (left). Forty-eight hours later, the animals were sacrificed. Their vasculatures were perfused with cold heparinized PBS, and the content of FGF1(ng/ml blood) was determined using an FGF1 ELISA kit. C. Contralateral (top) and postischemic (bottom) kidneys of an FGF1/c-fms mouse, 21 days after ischemia/reperfusion, during which period the animal was receiving water with doxycycline (660 mg/l). D. Sharp decrease of postischemic kidneys weight in FGF1/c-fms mice in comparison with control FVB animals (WT). Means and SEM are presented. Both types of mice received doxycycline in water throughout the experiment. E. Loss of tubular structures in the postischemic kidney of an FGF1/c-fms mouse. Representative hematoxylin and eosin stained paraffin sections of postischemic and contralateral kidneys of FGF1/c-fms and wild WT mice. Bar –80 µ.F. Postischemic/contralateral % ratio (mean and SEM) of kidney tubule density in FGF1/c-fms and WT mice. Numbers of epithelial tubular structures in ten ×10 objective fields were counted in postischemic and contralateral kidneys of six FGF1/c-fms and nine WT mice.

Like EC [37], macrophages and monocytes present a source of nonclassically secreted FGF1 and FGF2 in the organism [10]. To assess the effect of macrophage-derived FGF1 on postischemic kidney repair, we produced mice with monocyte/macrophage specific overexpression of FGF1. pTRE-Tight/FGF1 transgenic mice were crossed with rtTA/c-fms transgenic animals [30] that had been bred on an FVB background. Peritoneal macrophages that were obtained from the bi-transgenic FGF1/c-fms mice pretreated with doxycycline expressed FGF1:HA, while it was undetectable in macrophages obtained form the animals that did not receive doxycycline (Figure 7A). ELISA analysis demonstrated that doxycycline injection resulted in the appearance of FGF1 in the vasculature of FGF1/c-fms mice (Figure 7B). Thus, unlike FGF1/Tek mice, the cell type-specific expression of FGF1 in FGF1/c-fms animal depended on doxycycline stimulation. However, to equalize the mice for potential side effects of long-term doxycycline treatment, we choose to treat experimental (FGF1/c-fms) and control WT animals with doxycycline instead of comparing doxycycline-treated and untreated FGF1/c-fms mice. In unilateral kidney ischemia experiments, FGF1/c-fms and control WT two-month-old males were fed with doxycycline-containing water (660 mg/l), beginning 48 h before and up to day 21 after surgery. We found that like FGF1/Tek mice, FGF1/c-fms animals displayed a significant decrease of the weight of postischemic kidneys (Figure 7C,D) comparatively to WT animals. In addition, when compared to control mice the postischemic kidneys of FGF1/c-fms mice were characterized by enhanced hyperplasia of interstitial cells and significantly stronger loss of tubular structures (Figure 7E,F). Thus, similar to EC-derived, monocyte/macrophage-derived FGF1 can attenuate the postischemic kidney recovery.

## Discussion

Mice transgenically expressing FGF1 in EC are, to our knowledge, the first animal model used to study nonclassical protein export, its regulation and biological effects in vivo. We found that although FGF1/Tek mice displayed normal fertility, development, and phenotype (including normal kidney histology), they responded to ischemia/reperfusion by massive fibrosis. It is interesting that while in absence of ischemia, FGF1/Tek mice exhibited release of FGF1 to the circulation, it did not result in kidney pathologies. It is possible that continuous tissue stress following ischemic treatment stimulates FGF1 export from the basal surface of EC, which causes lasting proliferative stimulation of interstitial fibroblasts surrounding the capillaries. Alternatively, ischemia may induce the leakiness of the endothelial monolayer and thus diffusion of FGF1 released from the apical surface of EC to the exposed areas of the basal membrane. Tek (Tie2) promoter-driven expression is widely used for targeting transgene products to EC, particularly in the studies of the effects of FGF signaling in the cardiovascular systems [38]. However, there have been reports that Tek is also expressed in some subpopulations of macrophages [39]. Although our immunohistochemistry results show that EC is the locale where FGF1 is expressed in FGF1/Tek mice, we cannot exclude, that a portion of transgenic FGF1 could also be derived from macrophages.

The presence of numerous proliferating cells in the interstitium of postischemic kidneys of FGF1/Tek mice supports the hypothesis that ischemia stimulates FGF1 export from EC to surrounding structures. Recent work indicates that pericyte proliferation leads to the characteristic interstitial expansion seen in fibrosis following acute kidney injury [40], and FGF1 exported from EC may directly stimulate proliferation and migration of underlying pericytes. Additionally, FGF1 released to the interstitium of postischemic kidneys attracts neutrophils and macrophages that secrete the proinflammatory or mitogenic proteins: IL1, TGFbeta, ET-1, EGF, CTGF [41], [42], potentially promoting fibrosis. Thus, we propose that a complex and multifactorial cascade of events may cause the massive fibrosis and dysfunction seen in kidneys of mice with increased expression of nonclassically released FGF in EC.

Interestingly, Gerber et al. [43] recently reported that the knockout of FGFRL1, a decoy FGF receptor that binds the excess of FGFs, results in premature termination of metanephric kidney development, apparently because mesenchymo-epithelial transformation required for differentiation of tubular structures is inhibited by overabundant FGFs. The ubiquitous expression of FGF1 and FGF2 in the adult organism can reflect their role as latent stimulators of tissue repair, which become available when tissue stress induces their export. However, an excess of non-classically exported FGFs also can result in fibrosis and other pathologic processes. Indeed, the double knockout of FGF1 and FGF2 significantly decreases chemically induced liver fibrosis [44], while transgenic expression of FGF2 in cardiomyocytes exacerbated myocardial injury [45]. In addition, it is noteworthy that (i) genetically determined overexpression of FGF1 in the mesangial cells of kidneys correlates with hereditary hypertension [25]; (ii) FGF2 is implicated in pulmonary hypertension [26], (iii) FGF1 released by EC is a key stimulator of adipogenesis and thus can be involved in obesity [27].

The emerging hypothesis about the pathological potential of non-classically released FGFs, supported by the present work underlines the importance of pharmacological regulation of unconventional protein secretion. We found that taurine inhibited FGF1 export in vivo and rescued kidney repair in FGF1/Tek mice. The ability of taurine to suppress nonclassical protein export based on membrane stabilization may partially explain the beneficial effect of this small molecule in kidney fibrosis and other inflammatory diseases [34], [46], [47], [48].

In additional experiments using FGF1/c-fms mice, we found that FGF1 derived from macrophages can also induce the abnormal postischemic recovery of the kidney characterized by the loss of tubular structures and hyperplasia of interstitial cells. This effect is apparently due to the well-documented macrophage invasion into postischemic kidneys [49]. The ischemia efficiency may vary from experiment to experiment and that explains the difference in tubular structures loss in ischemic WT kidneys between Figures 3E and 7F. However, the trend of drastic exacerbation of postischemic damage in FGF1 transgenic animals is maintained.

We anticipate that this first study of FGF1 export in vivo and its effects on organ repair will increase the understanding of the biological effects of non-classical protein secretion, elucidation of its molecular mechanisms, and development of efficient methods of its regulation.

## Supporting Information

Figure S1 **Anti-CD31/PECAM immunoperoxidase staining of postischemic kidneys of WT and FGF1/Tek mice.** Three weeks after ischemia. Hematoxylin counterstaining. Bar 20 µ.
(TIFF)
