# Supplementary figures and images for: Transgenic Expression of Nonclassically Secreted FGF Suppresses Kidney Repair
Source: PLoS One. 2012 May 14;7(5):e36485. doi: 10.1371/journal.pone.0036485 (PMC3351418; doi:10.1371/journal.pone.0036485)

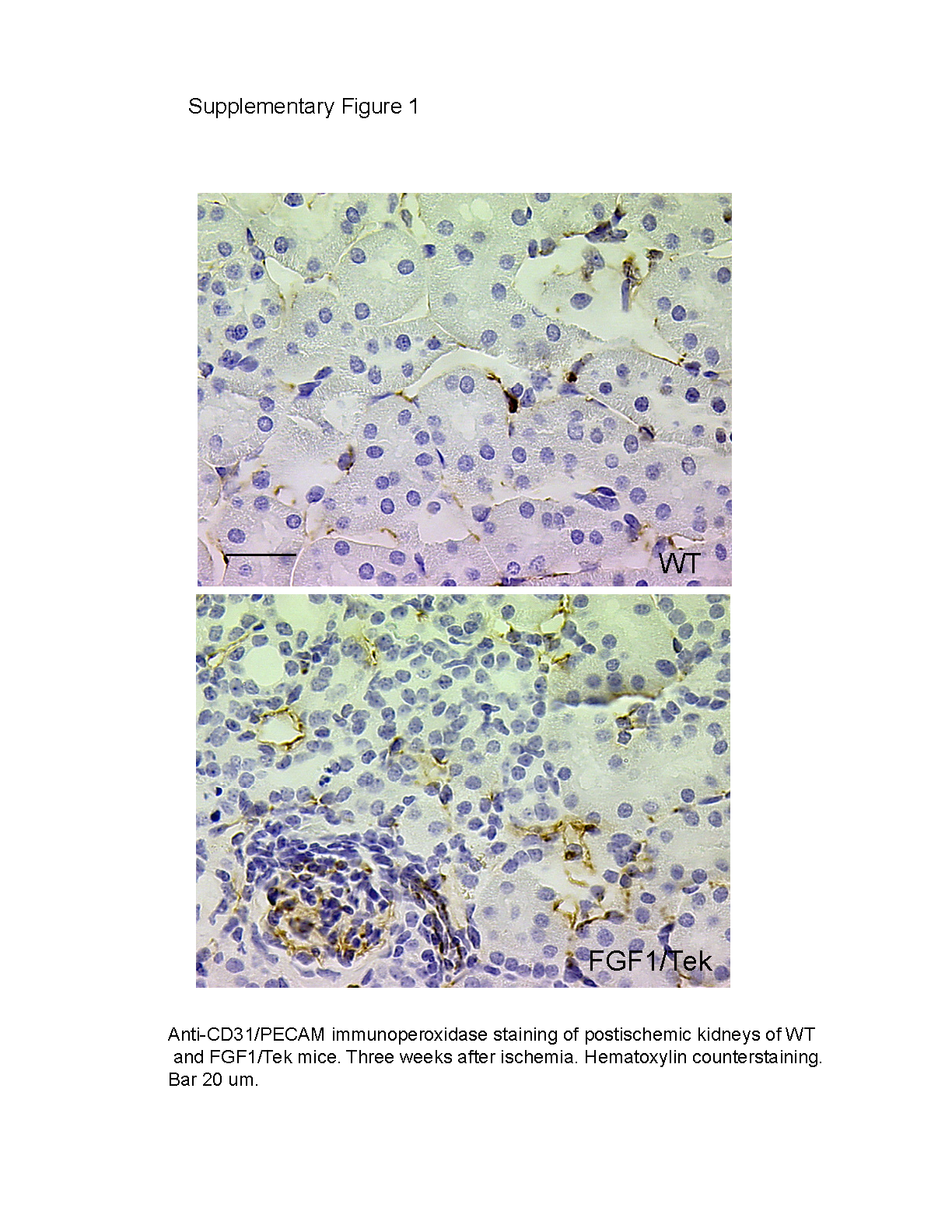

Supplement: Figure S1 — Anti-CD31/PECAM immunoperoxidase staining of postischemic kidneys of WT and FGF1/Tek mice. Three weeks after ischemia. Hematoxylin counterstaining. Bar 20 µ. (TIFF) [file pone.0036485.s001.tiff]
